# Association Between Orthostatic Hypotension With Coronary Slow Flow in Patients With Chest Pain: A Single Center Experience

**DOI:** 10.1002/clc.70050

**Published:** 2024-11-18

**Authors:** Lijun Han, Meng Li, Wenting Xie, Jianran Lu, Liang Yu, Xinying Liu, Na Lv, Lulu Zhang, Yan Zhang, Yanan Liu, Yanrong Li

**Affiliations:** ^1^ Department of cardiology and macrovascular disease Beijing Tiantan Hospital, Capital Medical University Beijing China; ^2^ Department of laboratory Beijing Tiantan Hospital, Capital Medical University Beijing China

**Keywords:** chest pain, coronary angiography, coronary slow flow, orthostatic hypotension, risk factors

## Abstract

**Background:**

Orthostatic hypotension (OH) is associated with different cardiovascular diseases, however, the association between OH and coronary slow flow (CSF) has never been evaluated before.

**Materials and Methods:**

Chest pain patients who underwent coronary angiography (CAG) and with normal coronary arteries in our department from January 1st, 2022 to August 31st, 2023 were retrospectively enrolled. Patients were divided into the CSF group and the normal blood flow (NBF) group. Relative clinical information, laboratory test results as well as the results of CAG were collected and analyzed. Both uni‐variable and multi‐variable logistic regression analyses were used to evaluate the association between OH and CSF in these patients.

**Results:**

Four thousand six hundred and twenty‐seven patients underwent CAG and 655 patients had normal coronary arteries. In which, sixty‐nine patients were diagnosed with CSF while 586 patients were diagnosed with NBF. Uni‐variable analysis revealed that higher body weight index, faster heart rate in sitting position, accompanied with chronic kidney disease, did not take Antidiabetic therapy, higher level of aspartate transaminase, uric acid, triglyceride, total cholesterol, ApoB1, low‐density lipoprotein cholesterol, homocysteine, B‐type natriuretic peptide as well as OH are the risk factors for CSF in these patients. Multi‐variable logistic regressing analysis further demonstrated that OH was the independent risk factor for predicting CSF in these patients.

**Conclusions:**

Our finding suggests OH might be a useful predictor for CSF in patients with chest pain but normal coronary arteries.

## Graphical abstract

In this retrospective study, 655 chest pain patients who underwent coronary angiography (CAG) and with normal coronary arteries were divided into coronary slow flow (CSF) group and normal blood flow group (NBF). After adjusting other co‐variables, orthostatic hypotension (OH) was associated with increased risk of CSF (OR:2.94, 95%CI:1.58‐5.45).

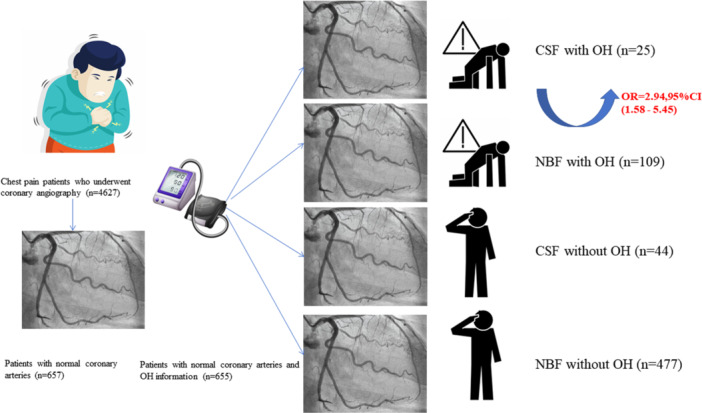

## 1 Background

Coronary slow flow (CSF) is characterized by a prolonged filling process of the coronary vasculature observed during coronary angiography (CAG) when patients exhibit a reduced Thrombolysis in Myocardial Infarction (TIMI) flow grade of less than 2 or an increased corrected TIMI frame count (CTFC) exceeding 27 frames in at least one coronary artery [1]. Studies have reported that 5.5%–34% of patients undergoing CAG are diagnosed with CSF [2]. A common occurrence in CAG, CSF has been linked to myocardial ischemia, arrhythmia, and even sudden cardiac death [3, 4, 5].

Orthostatic hypotension (OH) is defined as a significant drop in blood pressure within 3 min of standing up and is prevalent in clinical practice, particularly among patients with cardiovascular conditions [6]. OH has been associated with various cardiovascular diseases, such as atrial fibrillation, heart failure, and coronary artery disease (CAD) [7, 8, 9]. However, the relationship between OH and CSF has not been previously explored.

This retrospective study aims to investigate the connection between OH and CSF in patients experiencing chest pain with normal coronary arteries.

## 2 Materials and Methods

### 2.1 Study Design and Participants

This retrospective study was performed at Beijing Tiantan Hospital, Capital Medical University. Patients with chest pain who underwent CAG in our department from January 1st, 2022 to August 31st, 2023 were enrolled. Patients who were younger than 18 years old, patients who had stenosis coronary artery, patients who had percutaneous coronary intervention (PCI) or coronary artery bypass surgery (CABG) before, patients with severe hepatic or end‐stage renal disease (eGFR < 30 ml/min or on dialysis), patients who have neurological disorders and another disease like autoimmune disease and take medications which might influence the development of OH, patients who could not finish the measurement of OH and patients with acute myocarditis, coronary spasm, cardiomyopathy, coronary dissection were also excluded.

The baseline demographic information as well as prior medical history and medications were retrieved from electrical medical record system. Hypertension was defined as systolic blood pressure ≥ 140 mmHg and/or diastolic blood pressure ≥ 90 mmHg for at least two blood pressure measurements or on current anti‐hypertensive therapy. Diabetes mellitus was defined as a blood sugar level ≥ 7 mmol/L in the fasting state or on current Antidiabetic therapy. OH is defined as a reduction of at least 20 mmHg in systolic blood pressure or a reduction of at least 10 mmHg in diastolic blood pressure when patients change status from sitting to standing.

The laboratory test results including the results of the CAG were also retrieved from the electronic medical record system. Blood samples were analyzed by Sysmex BC‐6800 automatic blood analyzer (Mindray Bio‐Medical Electronics Co., Ltd, Shenzhen, China); biochemical function (liver function, kidney function, lipid profile, blood glucose, etc.) and cardiac enzyme was analyzed by Hitachi LABOSPECT 008AS biochemical analyzer (Hitachi High‐Tech Co., Tokyo, Japan). The value of left ventricular ejection fraction (LVEF) was evaluated by trained professionals using Simpson's method. The results of the CAG were evaluated by two or three trained cardiologists who were blind to the clinical and laboratory results. The CAG was performed using the Judkins technique via the femoral or radial artery in 15 frame/second mode. The evaluation of coronary blood flow was performed by using corrected TIMI frame count (CTFC), which in short is the number of cineframes required for contrast to fulfill the distal coronary landmarks at 30 frames/s. Since the LAD is longer than other arteries, the TIMI frame count was divided by 1.7 to calculate the CTFC for LAD. All the patients with CTFC > 27 in at least one coronary artery were divided into the CSF group.

Ethics approval was granted by Beijing Tiantan Hospital, Capital Medical University.

### 2.2 Statistical Analysis

Categorical variables were presented as numbers and frequencies (percentages) and were compared using the chi‐square test or exact Fisher test, as appropriate. Continuous variables were expressed as mean SD ± mean or median (quartile 1, quartile 3) based on normality assumption and were compared using the independent sample t‐test or Mann–Whitney test, as appropriate. Logistic analysis was performed by adjusting relative confounding factors with a *p* < 0.05. Data processing and analysis were performed using R version 4.3.0 (2023‐04‐21), along with Storm Statistical Platform (www.medsta.cn/software).

## 3 Result

**Figure 1:**
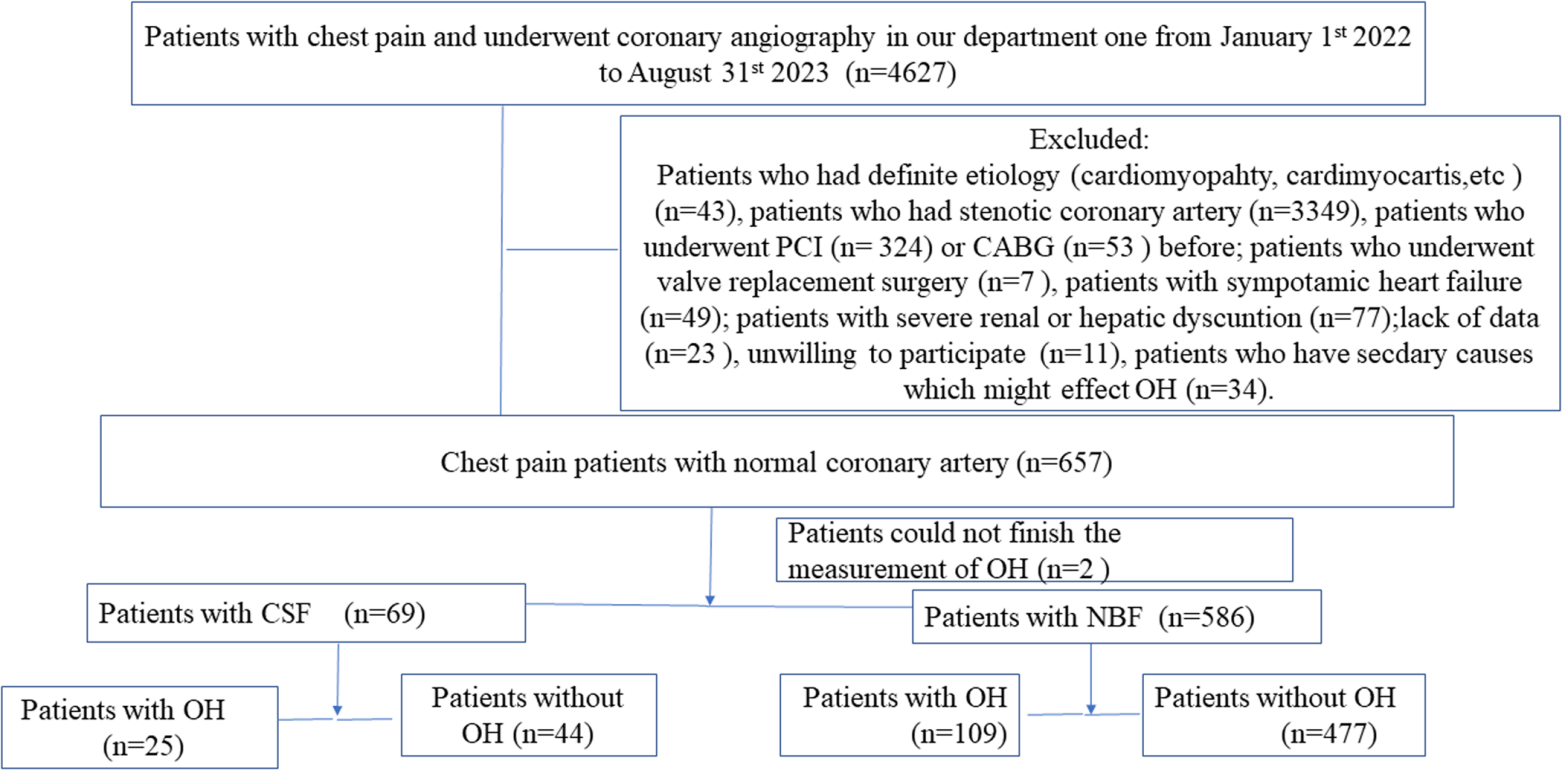
Study flow chart.

Four thousand six hundred and twenty‐seven patients underwent CAG and 655 patients had normal coronary arteries. In which, sixty‐nine patients were diagnosed with CSF while 586 patients were diagnosed with NBF. (Figure 1).

The comparison results of clinical parameters, baseline laboratory and echocardiography characteristics between patients with CSF and patients with NBF are shown in Table 1. In summary, CSF patients were higher in BMI [26.56 (25.35, 28.72) vs 25.56 (23.11, 27.68), *p* < 0.001] and more likely to have chronic kidney disease (CKD) (39.13% vs 20.31%, *p* = 0.004). Also, patients with CSF were less likely to receive Antidiabetic therapy (14.49% vs 27.13%, *p* = 0.023). Patients with CSF had a higher level of aspartate transaminase (AST) [21.10 (17.30, 25.80) vs 19.00 (16.30, 24.00), U/L, *p* = 0.026], higher level of uric acid (UA) [384.90 (308.90, 443.70) vs 337.20 (283.18, 402.45), mmol/L, *p* = 0.002] and a higher level of total triglyceride (TG) [1.66 (1.12,2.45) vs 1.39 (1.01,2.07), mmol/L, *p* = 0.017), higher level of total cholesterol(TC) [3.86 (3.32,4.63) vs 3.63 (3.12,4.39), mmol/L, *p* = 0.016], higher level of low‐density lipoprotein cholesterol (LDL‐C) [2.18 (1.80,2.99) vs 1.94 (1.52,2.60), mmol/L, *p* = 0.006], higher level of ApoB1 [0.76 (0.66,0.95) vs 0.70 (0.58, 0.86), mmol/L, *p* = 0.016], higher level of homocysteine (HCY) [14.13 (11.21,20.86) vs 12.28 (9.98,15.75), mmol/L, *p* < 0.001], higher level of B‐type natriuretic peptide (BNP) [241.70 (166.90, 329.60) vs 42.45 (20.18, 97.87), pg/mL, *p* < 0.001]. The CTFC values of the major coronary arteries were significantly different between the two groups.

**Table 1:** Baseline demographic, clinical characteristics, laboratory and angiographic characteristics of the study population.

Variables	All patients (*n* = 655)	NBF patients (*n* = 586)	CSF patients (*n* = 69)	Statistic	*p*
Age, M (Q₁, Q₃)	64.00 (57.00, 70.00)	64.00 (57.00, 70.75)	64.00 (57.00, 69.00)	Z = −0.77	0.439
BMI, M (Q₁, Q₃)	25.71 (23.26, 27.75)	25.56 (23.11, 27.68)	26.56 (25.35, 28.72)	Z = −3.32	**< 0.001**
Gender, n (%)				χ² = 0.93	0.335
Female	224 (34.20)	204 (34.81)	20 (28.99)		
Male	431 (65.80)	382 (65.19)	49 (71.01)		
Hypertension, n (%)				χ² = 0.37	0.544
No	211 (32.21)	191 (32.59)	20 (28.99)		
Yes	444 (67.79)	395 (67.41)	49 (71.01)		
DM, n (%)				χ² = 2.68	0.102
No	396 (60.46)	348 (59.39)	48 (69.57)		
Yes	259 (39.54)	238 (40.61)	21 (30.43)		
Dyslipidemia, n (%)				χ² = 0.66	0.415
No	209 (31.91)	184 (31.40)	25 (36.23)		
Yes	446 (68.09)	402 (68.60)	44 (63.77)		
Previous stroke, n (%)				χ² = 1.22	0.269
No	535 (81.68)	482 (82.25)	53 (76.81)		
Yes	120 (18.32)	104 (17.75)	16 (23.19)		
Previous myocardial infarction, n (%)				χ² = 2.10	0.147
No	560 (85.50)	497 (84.81)	63 (91.30)		
Yes	95 (14.50)	89 (15.19)	6 (8.70)		
Chronic kidney disease, n (%)				χ² = 12.63	**<0.001**
No	509 (77.71)	467 (79.69)	42 (60.87)		
Yes	146 (22.29)	119 (20.31)	27 (39.13)		
Smoke, n (%)				χ² = 0.04	0.845
No	382 (58.32)	341 (58.19)	41 (59.42)		
Yes	273 (41.68)	245 (41.81)	28 (40.58)		
Alcohol assumption, n (%)				χ² = 0.30	0.582
No	446 (68.09)	397 (67.75)	49 (71.01)		
Yes	209 (31.91)	189 (32.25)	20 (28.99)		
Premature CHD family history, n (%)				χ² = 0.17	0.677
No	519 (79.24)	463 (79.01)	56 (81.16)		
Yes	136 (20.76)	123 (20.99)	13 (18.84)		
Antiplatelet therapy, n (%)				—	0.495
No	261 (39.69)	237 (40.27)	24 (34.78)		
Yes	394 (60.15)	349 (59.56)	45 (65.22)		
Nitrogen, n (%)				χ² = 0.04	0.850
No	415 (63.36)	372 (63.48)	43 (62.32)		
Yes	240 (36.64)	214 (36.52)	26 (37.68)		
RASSi, n (%)				χ² = 0.49	0.482
No	392 (59.85)	348 (59.39)	44 (63.77)		
Yes	263 (40.15)	238 (40.61)	25 (36.23)		
Beta blocker, n (%)				χ² = 0.00	0.952
No	311 (47.48)	278 (47.44)	33 (47.83)		
Yes	344 (52.52)	308 (52.56)	36 (52.17)		
CCB, n (%)				χ² = 0.77	0.380
No	439 (67.02)	396 (67.58)	43 (62.32)		
Yes	216 (32.98)	190 (32.42)	26 (37.68)		
Antidiabetic therapy, n (%)				χ² = 5.15	**0.023**
No	486 (74.20)	427 (72.87)	59 (85.51)		
Yes	169 (25.80)	159 (27.13)	10 (14.49)		
WBC, M (Q₁, Q₃)	6.54 (5.31, 7.66)	6.55 (5.40, 7.68)	6.20 (4.95, 7.54)	Z = −1.71	0.088
RBC, M (Q₁, Q₃)	4.47 (4.11, 4.75)	4.46 (4.11, 4.76)	4.50 (4.17, 4.70)	Z = −0.13	0.896
PLT, M (Q₁, Q₃)	217.00 (182.00, 255.00)	219.00 (184.00, 256.00)	208.00 (160.00, 249.00)	Z = −1.96	0.050
ALT, M (Q₁, Q₃)	19.65 (14.30, 28.05)	19.40 (14.30, 27.70)	22.10 (15.10, 31.60)	Z = −1.43	0.153
AST, M (Q₁, Q₃)	19.10 (16.30, 24.20)	19.00 (16.30, 24.00)	21.10 (17.30, 25.80)	Z = −2.22	**0.026**
ALB, M (Q₁, Q₃)	39.60 (37.90, 41.35)	39.60 (37.90, 41.30)	39.40 (37.50, 41.40)	Z = −0.78	0.438
TB, M (Q₁, Q₃)	10.86 (8.18, 13.95)	10.70 (8.08, 13.80)	11.45 (8.52, 15.05)	Z = −1.16	0.245
Glucose, M (Q₁, Q₃)	5.43 (4.84, 6.41)	5.47 (4.87, 6.48)	5.18 (4.77, 5.83)	Z = −1.33	0.182
BUN, M (Q₁, Q₃)	5.60 (4.70, 6.70)	5.60 (4.70, 6.70)	5.90 (4.80, 6.80)	Z = −0.39	0.694
Creatinine, M (Q₁, Q₃)	66.60 (55.80, 77.40)	66.80 (55.50, 77.30)	65.30 (58.90, 79.00)	Z = −0.23	0.821
eGFR, M (Q₁, Q₃)	100.82 (88.20, 110.25)	100.96 (87.75, 110.32)	99.33 (91.83, 108.06)	Z = −0.24	0.812
UA, M (Q₁, Q₃)	342.20 (286.05, 407.70)	337.20 (283.18, 402.45)	384.90 (308.90, 443.70)	Z = −3.12	**0.002**
TG, M (Q₁, Q₃)	1.42 (1.01, 2.08)	1.39 (1.01, 2.07)	1.66 (1.12, 2.45)	Z = −2.38	**0.017**
TC, M (Q₁, Q₃)	3.66 (3.13, 4.44)	3.63 (3.12, 4.39)	3.86 (3.32, 4.63)	Z = −2.41	**0.016**
HDL‐C, M (Q₁, Q₃)	1.13 (0.98, 1.32)	1.13 (0.98, 1.31)	1.16 (1.01, 1.33)	Z = −0.63	0.526
LDL‐C, M (Q₁, Q₃)	1.98 (1.55, 2.65)	1.94 (1.52, 2.60)	2.18 (1.80, 2.99)	Z = −2.74	**0.006**
ApoA1, M (Q₁, Q₃)	1.29 (1.16, 1.46)	1.29 (1.16, 1.46)	1.31 (1.15, 1.44)	Z = −0.03	0.976
ApoB1, M (Q₁, Q₃)	0.70 (0.59, 0.87)	0.70 (0.58, 0.86)	0.76 (0.66, 0.95)	Z = −2.42	**0.016**
HCY, M (Q₁, Q₃)	12.40 (10.09, 16.30)	12.28 (9.98, 15.75)	14.13 (11.21, 20.86)	Z = −3.45	**< 0.001**
d‐Dimer, M (Q₁, Q₃)	0.46 (0.34, 0.60)	0.45 (0.33, 0.60)	0.48 (0.39, 0.69)	Z = −1.86	0.063
HbA1C, M (Q₁, Q₃)	6.20 (5.80, 7.00)	6.20 (5.80, 7.00)	6.10 (5.80, 6.60)	Z = −1.28	0.200
BNP, M (Q₁, Q₃)	49.60 (22.70, 129.95)	42.45 (20.18, 97.87)	241.70 (166.90, 329.60)	Z = −10.86	**< 0.001**
LVEF, M (Q₁, Q₃)	52.00 (47.00, 60.00)	52.00 (47.00, 60.00)	51.00 (46.00, 60.00)	Z = −0.94	0.350
CTFC of Coronary Arteries					
LAD, M (Q₁, Q₃)	34.00 (30.60, 37.40)	34.00 (30.60, 37.40)	40.80 (37.40, 49.30)	Z = −9.73	**< 0.001**
LCX, M (Q₁, Q₃)	21.00 (18.00, 24.00)	20.00 (18.00, 23.00)	26.00 (20.00, 29.00)	Z = −7.12	**< 0.001**
RCA, M (Q₁, Q₃)	21.00 (19.00, 24.00)	21.00 (19.00, 24.00)	29.00 (25.00, 30.00)	Z = −10.43	**< 0.0001**

Abbreviations: ALT, alanine aminotransferase; Apo, apolipoprotein; AST, aspartate transaminase; BMI, Body Mass Index; BNP, B‐type natriuretic peptide; CAD, coronary artery disease; CCB, calcium channel blocker; CTFC; corrected TIMI frame count; eGFR, estimated glomerular filtration rate; HbA1C, Glycosylated Hemoglobin, Type A1C; HCY, homocysteine; HDL‐C, high‐density lipoprotein cholesterol; LAD, left anterior descending artery; LCX, left circunflex artery; LDL‐C, low‐density lipoprotein cholesterol; LVEF, left ventricular ejection fraction; RASSi, angiotensin‐converting enzyme inhibitor; RCA, right coronary artery; TC, total cholesterol; TG, Triglyceride; TSH, thyroid stimulating hormone; UA, uric acid.

**Figure 2:**
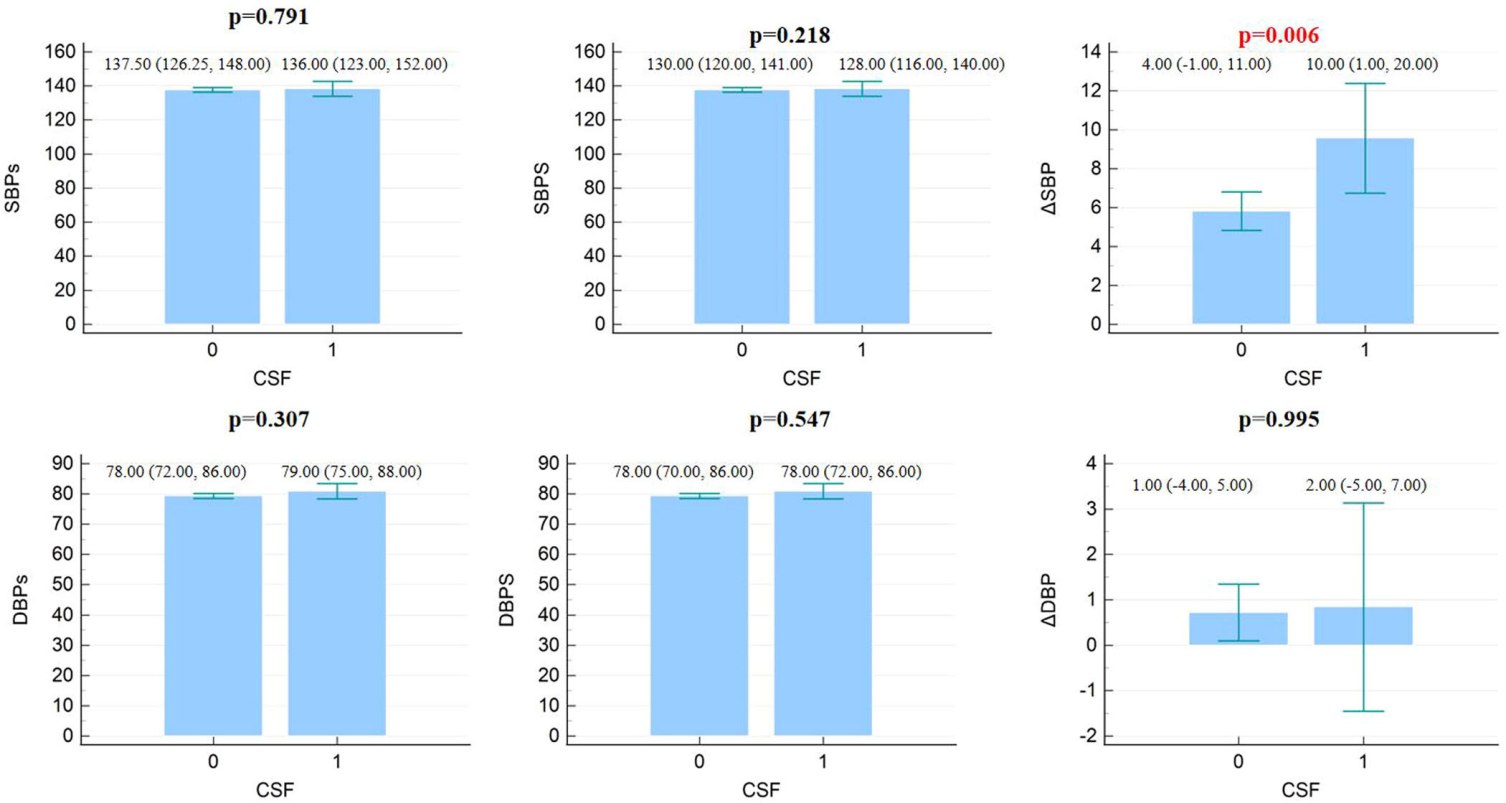
Comparison result of blood pressure parameters in patients with CSF and NBF.

The comparison results of blood pressure and heart rate parameters between patients with CSF and patients with NBF are shown in Table 2. In summary, the heart rate, as well as the systolic/diastolic blood pressure, were all comparable between the two groups when the patients were in a sitting position. In patients with CSF, the heart rate in a standing position was lower than in patients with NBF [72.00 (70.00, 78.00) vs 72.00 (70.00, 74.00), bpm, *p* = 0.005]. Also, the systolic and diastolic blood pressure changes were larger in CSF patients when compared with patients with NBF (10.00 (1.00, 20.00) vs 4.00 (−1.00, 11.00), mmHg, *p* = 0.006; 2.00 (−5.00, 7.00) vs 1.00 (−4.00, 5.00), mmHg, *p* < 0.001, respectively) (Figure 2). Also, CSF patients were more likely to have OH when compared with patients with NBF (36.23% vs 18.60%, *p* < 0.001).

**Table 2:** Comparison of the blood pressure and heart rate parameters of the study population.

Variables	All patients (*n* = 655)	NBF patients (*n* = 585)	CSF patients (*n* = 69)	Statistic	*p*
Sitting heart rate, M (Q₁, Q₃)	72.00 (70.00, 77.00)	72.00 (70.00, 78.00)	72.00 (70.00, 74.00)	Z = −1.67	0.095
Standing heart rate, M (Q₁, Q₃)	72.00 (70.00, 78.00)	72.00 (70.00, 78.00)	72.00 (70.00, 74.00)	Z = −2.80	**0.005**
Sitting SBP, M (Q₁, Q₃)	137.00 (126.00, 149.00)	137.50 (126.25, 148.00)	136.00 (123.00, 152.00)	Z = −0.27	0.791
Sitting DBP, M (Q₁, Q₃)	78.00 (72.00, 86.00)	78.00 (72.00, 86.00)	79.00 (75.00, 88.00)	Z = −1.02	0.307
Standing SBP, M (Q₁, Q₃)	130.00 (120.00, 141.00)	130.00 (120.00, 141.00)	128.00 (116.00, 140.00)	Z = −1.23	0.218
Standing DBP, M (Q₁, Q₃)	78.00 (70.00, 86.00)	78.00 (70.00, 86.00)	78.00 (72.00, 86.00)	Z = −0.60	0.547
ΔSBP, M (Q₁, Q₃)	4.00 (−1.00, 12.00)	4.00 (−1.00, 11.00)	10.00 (1.00, 20.00)	Z = −2.75	**0.006**
ΔDBP, M (Q₁, Q₃)	1.00 (−4.00, 5.00)	1.00 (−4.00, 5.00)	2.00 (−5.00, 7.00)	Z = −0.01	0.995
OH, n (%)				χ² = 11.79	**< 0.001**
No	521 (79.54)	477 (81.40)	44 (63.77)		
Yes	134 (20.46)	109 (18.60)	25 (36.23)		

Abbreviations: DBP, diastolic blood pressure; OH, orthostatic hypotension; SBP, systolic blood pressure; Δ, blood pressure in sitting position minus blood pressure in standing position.

We also performed multivariable logistic regression analysis to evaluate the association between OH and CSF. In summary, OH and lower heart rate in standing position, higher level of HCY, and BNP, accompanied by CKD as well as not receiving Antidiabetic therapy were the independent risk factors for CSF (Table 3).

**Table 3:** Uni‐variable and multi‐variable logistic analysis in predicting CSF.

	Uni‐variable analysis					Multi‐variable analysis				
Variables	β	S.E	Z	*P*	OR (95%CI)	β	S.E	Z	*P*	OR (95%CI)
Standing heart rate	−0.05	0.02	−2.55	**0.011**	0.95 (0.92 ~ 0.99)	−0.04	0.02	−2.31	**0.021**	0.96 (0.92 ~ 0.99)
AST	0.01	0.01	1.27	0.203	1.01 (1.00 ~ 1.02)	0.00	0.01	0.30	0.764	1.00 (0.99 ~ 1.02)
UA	0.01	0.00	3.00	**0.003**	1.01 (1.01 ~ 1.01)	0.00	0.00	0.88	0.378	1.00 (1.00 ~ 1.00)
TG	0.15	0.09	1.61	0.106	1.16 (0.97 ~ 1.40)	0.29	0.15	1.91	0.057	1.34 (0.99 ~ 1.80)
TC	0.30	0.12	2.43	**0.015**	1.36 (1.06 ~ 1.73)	−0.21	0.47	−0.44	0.660	0.81 (0.33 ~ 2.03)
LDL	0.42	0.15	2.85	**0.004**	1.52 (1.14 ~ 2.03)	0.77	0.61	1.25	0.211	2.15 (0.65 ~ 7.16)
ApoB1	1.36	0.54	2.55	**0.011**	3.91 (1.37 ~ 11.17)	−0.69	1.58	−0.44	0.662	0.50 (0.02 ~ 11.11)
HCY	0.03	0.01	3.42	**< 0.001**	1.03 (1.01 ~ 1.05)	0.02	0.01	2.27	**0.023**	1.02 (1.01 ~ 1.05)
BNP	0.01	0.00	6.77	**< 0.001**	1.01 (1.01 ~ 1.01)	0.01	0.00	5.94	**< 0.001**	1.01 (1.01 ~ 1.01)
CKD										
No					1.00 (Reference)					1.00 (Reference)
Yes	0.93	0.27	3.46	**< 0.001**	2.52 (1.49 ~ 4.26)	0.81	0.31	2.58	**0.010**	2.24 (1.21 ~ 4.14)
Antidiabetic therapy										
No					1.00 (Reference)					1.00 (Reference)
Yes	−0.79	0.35	−2.22	**0.026**	0.46 (0.23 ~ 0.91)	−0.87	0.41	−2.12	**0.034**	0.42 (0.19 ~ 0.94)
OH										
No					1.00 (Reference)					1.00 (Reference)
Yes	0.91	0.27	3.35	**< 0.001**	2.49 (1.46 ~ 4.24)	1.08	0.31	3.42	**< 0.001**	2.94 (1.58 ~ 5.45)

Abbreviations: AST, aspartate transaminase; BNP, B‐type natriuretic peptide; C, low‐density lipoprotein cholesterol; CI, Confidence Interval; CKD, chronic kidney disease; LDL‐HCY, homocysteine; OR, Odds Ratio; TC, total cholesterol; TG, triglyceride; UA, uric acid.

## 4 Discussion

In this retrospective study, we examined the relationship between orthostatic hypotension (OH) and coronary slow flow (CSF) in patients presenting with chest pain and normal coronary arteries. To the best of our knowledge, this is the first study to investigate this particular association.

CSF, initially documented in 1972, demonstrates a high prevalence in patients who have undergone coronary angiography (CAG) [10]. The clinical spectrum of CSF ranges from asymptomatic to potentially life‐threatening conditions, posing ongoing challenges for long‐term clinical management due to recurrent chest pain and hospital readmissions in select cases [11, 12, 13]. While the exact mechanisms underlying CSF remain under investigation, there is a consensus that endothelial dysfunction, microvascular disease, systemic inflammation, and atherosclerosis play roles in its development [14, 15, 16, 17]. Previous research has identified various clinical risk factors independently linked to CSF, including being young, male, a current smoker, having multiple cardiovascular comorbidities, and possessing a higher BMI [11]. Novel indicators such as the triglyceride glucose index, triglyceride/high‐density lipoprotein cholesterol ratio, and systemic immune‐inflammation index have also shown associations with CSF [16, 18, 19]. Given the complexity of these parameters, there is a significant need to identify a straightforward predictor for CSF in clinical settings.

In our study, we observed that patients with CSF were more likely to have elevated level of homocysteine (HCY), brain natriuretic peptide (BNP), standing heart rate, also the proportion of patients with chronic kidney disease (CKD), patients who received antidiabetic therapy and patients with OH were higher.

The association between homocysteine and CSF has been widely discussed. In a retrospective study by Yang S, et al, the level of homocysteine was positively correlated with CSF after adjustment other parameters [17]. Demirci E, et al enrolled 23 patients with CSF and 25 patients with NBF, the plasma homocysteine level was significantly higher in patients with CSF compared with patients with NBF (16.2 ± 7.6 vs. 12.2 ± 2.2 μM/L; *p* = 0.023) [18]. This finding is actually consisted with a meta‐analysis enrolled 13 studies with 625 CSF patients and 550 NBF patients and this meta‐analysis confirmed that elevated Hcy levels was strongly associated with CSF [19]. The underlying mechanism of homocysteine in CSF is still unclear. Hyperhomocysteinemia is a well‐accepted risk factor for cardiovascular diseases which could compromise the endothelial function possibly by reducing the production of vasodilator (nitric oxide) and increasing oxidative stress [18, 20, 21, 22].

The study which evaluate the association between BNP and CSF was scarce. In a cardiac magnetic resonance imaging (CMR)‐based study, the relationship between the presence of myocardial fibrosis and NT‐proBNP levels in patients with CSF in the left anterior descending coronary artery (LAD) were evaluated. Nineteen CSF patients and 16 NBF patients were enrolled in the study, the NT‐proBNP levels were higher in CSF patients with scar tissue in CMR compared with NBF patients [23]. This finding aligns with a study conducted by Yurtdaş M, which reported elevated baseline levels of NT‐Pro‐BNP concentrations in patients with CSF [24]. The reason of CSF patients having higher level of BNP may be related to heart dysfunction caused by scar tissue and potential microvascular or epicardial coronary artery dysfunction according to the finding of these studies.

Our study found that CKD was associated with CSF, as a matter of fact, there was no other similar studies as we known. The mechanism of the association between CKD and CSF is unclear but the endothelial dysfunction, oxidative stress and inflammation state in patients with CKD might explain this phenomenal [25, 26, 27].

The key finding of our study highlights OH as an independent risk factor for CSF. While the relationship between OH and other cardiovascular diseases has been extensively studied, the link between OH and CSF has not been previously explored. Our analysis, both uni‐variable and multi‐variable, identifies OH as a standalone risk factor for CSF presence. The exact underlying mechanism connecting OH and CSF remains unclear. Nonetheless, given the established mechanisms of CSF involving atherosclerosis, inflammation, microvascular function, and autonomic dysfunction, it is plausible that the association between CSF and OH may be attributed to atherosclerosis, endothelial dysfunction, and autonomic dysfunction induced by OH [28, 29, 30].

Several limitations exist in our study. Firstly, being a single‐center retrospective study with a limited participant pool, further validation through a multi‐center prospective study with a larger cohort is necessary. Secondly, our study defines OH based on classical criteria, excluding patients with delayed OH, thus obscuring the clinical relevance of different types of OH in CSF development. Thirdly, the diagnosis of OH in our study relies on a simple standing test rather than a more precise device‐based tilt‐up test, warranting additional studies utilizing advanced equipment to confirm our findings. In summary, our study is the first to establish an association between OH and CSF in patients presenting with chest pain, suggesting that OH could serve as a straightforward screening tool for CSF.

## Conflicts of Interest

The authors declare no conflicts of interest.

## Data Availability

The data that support the findings of this study are available from the corresponding author upon reasonable request.
